# A Case Report of Epiglottitis in an Adult Patient

**DOI:** 10.21980/J8QM09

**Published:** 2022-01-15

**Authors:** Savannah Tan, Kyle Dornhofer, Allen Yang, Shadi Lahham, Lindsey C Spiegelman

**Affiliations:** * University of California, Irvine, Department of Emergency Medicine, Orange, CA

## Abstract

Epiglottitis is historically known to be a disease of childhood. However, since the implementation of the *Haemophilus influenzae* type B vaccination, there has been an increasing incidence within the adult population. This is a case report of a 36-year-old male who presented to the emergency department (ED) in severe respiratory distress with complaints of a sore throat, shortness of breath, odynophagia, dysphagia, and hoarseness. Physical exam revealed biphasic stridor, tachycardia, tachypnea, and an erythematous uvula. Imaging of his neck revealed findings consistent with epiglottitis containing nonspecific air. The patient was rapidly intubated, started on broad spectrum antibiotics and dexamethasone. He was admitted for intensive medical management with Otolaryngology consultation. This case report highlights the importance of rapid recognition and diagnosis of epiglottitis in an adult population to prevent morbidity and mortality.

**Topics:**

Epiglottitis, stridor, odynophagia, dysphagia, *Haemophilus influenzae*, group C *streptococci*, thumb-print sign, intubation.

**Figure:**
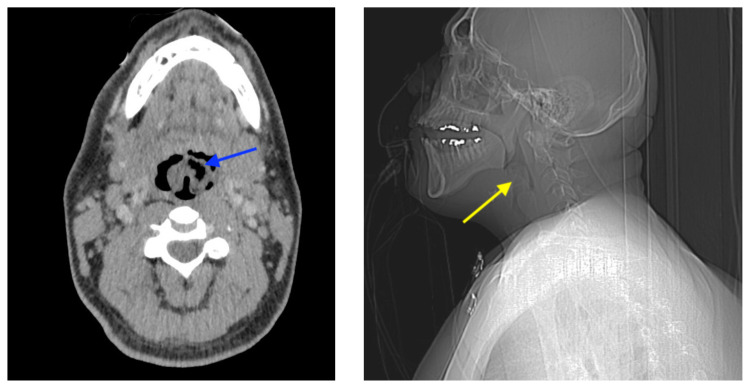
Sagittal CT Video Link: https://youtu.be/xUMQ2Kk8UeY

**Figure:**
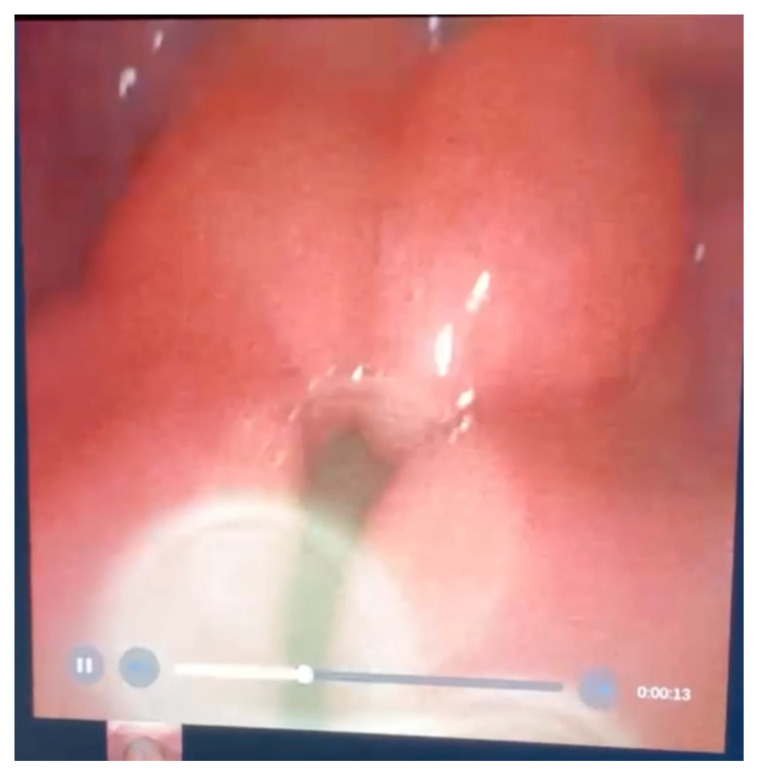
Video Laryngoscopy Link: https://youtu.be/C0CDbOWGCmE

## Brief introduction

Epiglottitis is a potentially life-threatening inflammation of the epiglottis and surrounding structures that results in signs and symptoms of acute and progressive airway obstruction. Though historically known to afflict children, epiglottitis has now become a disease of adults as a result of the widespread use of the *Haemophilus influenzae* (HiB) vaccine, which helped dramatically decrease the incidence in children.^1^ The mean annual incidence rate of epiglottitis in adults from 1996 to 2000 was 3.1 per 100,000 adults, and the mortality rate of adults diagnosed with epiglottitis ranges between 6 to 7%, which is significantly higher than the less than 1% mortality rate in children, due mainly to delays in recognition in adult patients, misdiagnosis, or inappropriate treatment.^2^,^3^ Serious consequences and fatal upper airway obstruction may occur without prompt diagnosis and treatment of acute epiglottitis. In this report, we highlight the signs and symptoms of epiglottitis, methods for diagnosis, and clinical management of the disease process in an adult patient.

## Presenting concerns and clinical findings

A 36-year-old male presented to the emergency department with complaints of a severe sore throat and shortness of breath that began in the morning. The patient was seen earlier that day at another emergency department with similar symptoms and was noted to be very anxious with minimal erythema on exam and oxygen saturation of 100% on room air. At the time, he was given one dose of hydroxyzine and discharged home with a diagnosis of pharyngitis and anxiety. Upon evaluation, he also reported throat tightness, difficulty swallowing, and voice changes that were constant and worsening since onset. Review of systems was also positive for headache. He denied tongue, lip, or mouth swelling, allergies to food, rash, itching, vomiting, or diarrhea. He was fully vaccinated and denied history of prior similar episodes. The patient’s past medical history was significant for diabetes, obesity, and primary syphilis. Past surgical and family history were noncontributory. On exam, the patient was found to be in severe apparent respiratory distress, drooling and intermittently spitting with a muffled voice. His uvula was mildly erythematous, with biphasic stridor noted on pulmonary exam. Vital signs were also notable for tachycardia, tachypnea, and an elevated blood pressure of 159/74 mmHg, with oxygen saturation of 98% on room air.

## Significant findings

At the time of presentation to the ED, laboratory results were significant for leukocytosis to 11.8 × 10^9^ white blood cells/L and a partial pressure of carbon dioxide of 52 mmHg on venous blood gas. Computed tomography (CT) of the soft tissue of the neck with contrast showed edematous swelling of the epiglottis and aryepiglottic fold with internal foci of gas (blue arrow) and partial effacement of the laryngopharyngeal airway and scattered cervical lymph nodes bilaterally (Figure 1). Findings were consistent with epiglottitis containing nonspecific air. Additionally, the pathognomonic “thumbprint sign” (yellow arrow) was found on lateral x-ray of the neck (Figure 2). The CT findings as shown in figure 3 illustrate lateral view of the swelling of the epiglottis, gas, and blockage of the airway.

## Patient course

Intravenous diphenhydramine, dexamethasone, and nebulized racemic epinephrine were initiated within minutes of the patient’s arrival. Following the diagnosis of epiglottitis by CT imaging and continued respiratory distress, the patient was transported to the resuscitation bay for emergency laryngoscopy, which confirmed severe epiglottic and arytenoid edema and showed pooling of secretions in the oropharynx. Emergent intubation was performed via a trans-nasal fiberoptic approach to address his severe continued respiratory distress, with some difficulty encountered when passing the epiglottis due to an extremely edematous epiglottis and narrowed larynx. The patient was then admitted to the medical intensive care unit (ICU) for initiation of broad-spectrum antibiotics including vancomycin, ceftriaxone, and clindamycin. Two days after admission, blood cultures reported no growth and bronchoscopy showed continued edema of the epiglottis with black discoloration of the left anterolateral surface and diffusely edematous airways with scattered, thick secretions throughout. A bronchoalveolar lavage (BAL) was performed in the right middle lobe, and microbiology labs including cell count, fungal, bacterial, acid-fast bacilli, and *Pneumocystis jiroveci* pneumonia (PJP) labs were sent. Given the findings of necrosis on bronchoscopy, the antibiotic regimen was switched to piperacillin-tazobactam for broad gram-negative rod and pseudomonas coverage, with continuation of vancomycin and addition of acyclovir, micafungin, and trimethoprim-sulfamethoxazole for empiric herpes simplex virus (HSV) and varicella-zoster virus (VZV) coverage, antifungal coverage, and PJP prophylaxis, respectively.

During the hospital course, the patient was found to be human immunodeficiency virus (HIV) positive with a CD4 count of 104 cells/μL. As a result, routine studies including serum cryptococcal antigen, toxoplasma IgG and IgM, histoplasma antigen, cytomegalovirus, toxoplasma, coccidioides screenings, and tuberculosis (TB) QuantiFERON blood labs were ordered. The patient was also started on Biktarvy therapy, an antiretroviral regimen consisting of bictegravir, emtricitabine and tenofovir alafenamide.

A repeat CT soft tissue of the neck with contrast was eventually performed and exhibited residual epiglottic edema with resolution of prior noted soft tissue gas. Upon repeat flexible laryngoscopy, edema of the epiglottis was redemonstrated without any visualized regions of necrosis. Cultures from the BAL revealed few beta-hemolytic group C *streptococci* and few yeasts, and hepatitis A IgG was found to be reactive. All other labs including histoplasma, cryptococcal, coccidioides, toxoplasma, cytomegalovirus, QuantiFERON TB, chlamydia, gonorrhoeae, hepatitis A IgM, hepatitis B core and surface antigen, and hepatitis C antibodies were negative. Given the improved findings on imaging, negative bronchoalveolar lavage acid-fast bacilli and pneumocystis pneumonia results and sputum cultures results, and decreased fraction of inspired oxygen (FiO2) requirements, the patient was extubated on hospital day seven to continuous positive airway pressure (CPAP) and transitioned to room air the day after. As the patient continued to improve, his antibiotics were transitioned from intravenous to oral medications, and he was discharged with plans to complete a 2-week total course of antibiotics and a follow up appointment with an HIV care clinic.

## Discussion

Epiglottitis is an infection of the epiglottis and supraglottic larynx that results in swelling and inflammation of these structures, with the potential to cause rapid respiratory compromise. Though a variety of organisms have been linked to epiglottitis, most cases result in negative sputum cultures and blood cultures with no organism definitively identified.^4^ In children, when an organism is isolated, the most common offending bacterium is H. *influenzae*; in adults, a more diverse microbiological etiology is often found. Epiglottitis in adults may be comprised of a variety of organisms including beta-hemolytic *streptococcus, Streptococcus pneumoniae, and Staphylococcus aureus*, among others.^4^,^5^ Consistent with these findings, this case report shows evidence of possible infection of epiglottitis caused by Group C *Streptococcus*, a beta-hemolytic *streptococcus* species.

Typical signs and symptoms of acute infection may include fever, sore throat, odynophagia, dysphagia, neck tenderness, stridor, drooling, and shortness of breath. Patients with epiglottitis may exhibit voice changes, such as hoarseness or refusal to talk due to pain, and may appear anxious. As the infection progresses and airway blockage becomes more severe, patients begin to exhibit worsening stridor and profound dyspnea that may result in a red flag symptom termed tripod positioning, indicating impending upper airway obstruction. The tripod sign refers to the classic positioning that a person takes when they are experiencing severe dyspnea: the patient leans forward on outstretched arms, neck extended, with maximal jaw thrust effort in order to move the inflamed and swollen epiglottic structures forward and thus open the narrowed airway.^6^ The gold standard for diagnosis of epiglottitis is through direct visualization of the edematous epiglottis by fiberoptic laryngoscopy. Lateral neck x-rays are also of utility with a sensitivity of 88%, with demonstration of the characteristic “thumb-print” sign indicative of the swelling seen in epiglottitis, but with less sensitivity than direct visualization.^7^,^8^

Critical consequences of epiglottitis can result if the infection is not promptly identified and diagnosed. Specifically, stridor, tachycardia, tachypnea, rapid onset of symptoms, accessory muscle use, and the thumb-print sign on lateral x-ray of the neck are significant indicators of imminent airway compromise with resulting rapid clinical deterioration.^9^ Other sequelae of acute epiglottitis include deep neck space infection, recurrent illness, and vocal granuloma. Once the clinical course has progressed to the point of severe respiratory distress, the patient should be treated as a medical and airway emergency. Emergent securing of the airway must be prioritized to prevent cardiopulmonary arrest. Prompt consultation with airway experts including otolaryngology and anesthesiology at the bedside is recommended. Intubation of a patient with acute epiglottitis should be performed under anesthesia with maintenance of spontaneous ventilation with the patient sitting upright to prevent acute airway obstruction from a supine position, with fiberoptic nasal intubation or rigid bronchoscopy using an endotracheal tube as the method of choice.^10^ As for medical management, empiric broad combination antibiotic therapy with a third-generation cephalosporin and vancomycin is usually recommended, with adjustments as culture sensitivities are identified for a total of at least 10 days.^6^ Routine vaccination for HiB is not recommended in adults, unless the patient is at high risk of contracting H. *influenzae* infection, as in those with a history of asplenia, immunodeficiency, immunosuppression from cancer chemotherapy, infection with HIV, or receipt of a hematopoietic stem cell transplant.^6^ In addition, corticosteroid use has been associated with a shorter ICU and overall length of hospitalization, with an average overall hospital course of 2.9 days as compared with 6.9 days for patients not treated with corticosteroids.^11^ Regardless of placement of an airway, patients are best monitored closely in the ICU for airway deterioration.

Although acute epiglottitis is now a relatively uncommon disease as a result of widespread vaccination efforts, suspicion, recognition, and diagnosis of epiglottic infection in adults remains vital in preventing rapid respiratory compromise and severe clinical decline in these patients. This case serves as an important reminder for Emergency Medicine physicians to evaluate for epiglottitis in patients presenting with odynophagia, dysphagia, and stridor, considering that these cases can swiftly become medical emergencies. Early identification and management of these presentations can prevent the need for intubation and possible serious sequelae of the disease process.

## Supplementary Information




